# Molecular analysis of the cumulus matrix: insights from mice with *O*-glycan-deficient oocytes

**DOI:** 10.1530/REP-14-0503

**Published:** 2015-05

**Authors:** Panayiota Ploutarchou, Pedro Melo, Anthony J Day, Caroline M Milner, Suzannah A Williams

**Affiliations:** Nuffield Department of Obstetrics and Gynaecology, Women's Centre, Level 3, John Radcliffe Hospital, University of Oxford, Oxford, OX3 9DU, UK; 1 Faculty of Life Sciences Michael, University of Manchester, Smith Building, Oxford Road, Manchester, M13 9PT, UK; 2 Wellcome Trust Centre for Cell-Matrix Research, Faculty of Life Sciences, University of Manchester, Michael Smith Building, Oxford Road, Manchester, M13 9PT, UK

## Abstract

During follicle development, oocytes secrete factors that influence the development of granulosa and cumulus cells (CCs). In response to oocyte and somatic cell signals, CCs produce extracellular matrix (ECM) molecules resulting in cumulus expansion, which is essential for ovulation, fertilisation, and is predictive of oocyte quality. The cumulus ECM is largely made up of hyaluronan (HA), TNF-stimulated gene-6 (TSG-6, also known as TNFAIP6), pentraxin-3 (PTX3), and the heavy chains (HCs) of serum-derived inter-α-inhibitor proteins. In contrast to other *in vivo* models where modified expansion impairs fertility, the cumulus mass of *C1galt1* Mutants, which have oocyte-specific deletion of core 1-derived *O*-glycans, is modified without impairing fertility. In this report, we used *C1galt1* Mutant (*C1galt1* ^*FF*^:ZP3*Cre*) and Control (*C1galt1* ^*FF*^) mice to investigate how cumulus expansion is affected by oocyte-specific deletion of core 1-derived *O*-glycans without adversely affecting oocyte quality. Mutant cumulus–oocyte complexes (COCs) are smaller than Controls, with fewer CCs. Interestingly, the CCs in Mutant mice are functionally normal as each cell produced normal levels of the ECM molecules HA, TSG-6, and PTX3. However, HC levels were elevated in Mutant COCs. These data reveal that oocyte glycoproteins carrying core 1-derived *O*-glycans have a regulatory role in COC development. In addition, our study of Controls indicates that a functional COC can form provided all essential components are present above a minimum threshold level, and thus some variation in ECM composition does not adversely affect oocyte development, ovulation or fertilisation. These data have important implications for IVF and the use of cumulus expansion as a criterion for oocyte assessment.

## Introduction

The process of follicle development begins with the activation of a quiescent primordial follicle and culminates with the ovulation of a single fertilisable egg. During the early stages of follicle development, the granulosa cells that surround the oocyte proliferate to form multiple layers of cells. As the follicle develops, antral fluid is deposited between the granulosa cells which facilitates the physical separation and differentiation of the granulosa cell population into mural granulosa cells (mGCs; which line the wall of the follicle) and cumulus cells (CCs) that are associated with the oocyte.

Prior to ovulation, an extracellular matrix (ECM) is assembled between the CCs leading to expansion of the cumulus mass that surrounds the oocyte. The expanded cumulus mass is believed to facilitate efficient capture of an ovulated egg by the oviductal fimbriae and transport into the oviduct (Chen *et al*. 1993, Tanghe *et al*. 2002). Furthermore, oocyte quality has been linked to the degree of cumulus expansion in humans (Ng *et al*. 1999). Therefore, ECM deposition and cumulus expansion are important for ovulation, fertilisation and implantation.

Ovulation is stimulated by the release of luteinising hormone (LH), which initiates two signalling events. First, mGCs secrete epidermal growth factor-like (EGF-L) peptides that bind to EGF receptors on CCs (Park *et al*. 2004). Secondly, also acting on CCs, the oocyte produces soluble growth factors termed as oocyte-secreted factors (OSFs) that are required for cumulus expansion in mice; these include members of the transforming growth factor beta (TGF-β) superfamily (e.g. GDF9 and BMP15) (Su *et al*. 2004, Dragovic *et al*. 2005, Peng *et al*. 2013). Binding of TGF-β ligands to cognate receptors on CCs results in the activation of signal transduction pathways mediated via either SMAD2/3 or SMAD1/5/8 (Knight & Glister 2006). Although OSFs have a central role in cumulus expansion in mice, a similar role has not been shown in mono-ovulatory species. Cow and ovine cumulus–oocyte complexes (COCs) undergo follicle-stimulating hormone-induced cumulus expansion *in vitro* in the absence of the oocyte, suggesting that OSFs are not vital for cumulus expansion in all species (Gilchrist *et al*. 2008, Varnosfaderani Sh *et al*. 2013). The role of OSFs in COC expansion in humans is currently unclear (Gilchrist *et al*. 2008).

Following the LH surge, the activation of EGF- and OSF-mediated signalling pathways induces CCs to express hyaluronan synthase 2 (HAS2) and synthesise the glycosaminoglycan hyaluronan (HA) (Fulop *et al*. 1997*a*), the major structural component of the viscoelastic cumulus ECM (Salustri *et al*. 1989). The organisation and stability of the cumulus matrix are dependent on cross linking of the HA polysaccharide, which is mediated by several proteins, including pentraxin-3 (PTX3), TSG-6 (which is the secreted protein product of TNFAIP6, hereafter referred to as TSG-6) and the heavy chains (HCs) of inter-α-inhibitor (IαI) and pre-α-inhibitor (PαI) (Sato *et al*. 2001, Zhuo *et al*. 2001, Fulop *et al*. 2003, Salustri *et al*. 2004, Scarchilli *et al*. 2007).

IαI and PαI are synthesised in the liver and transported in serum, but their size and charge cause them to be excluded from the follicle by the basal lamina (Hess *et al*. 1998). At ovulation, the LH surge initiates the breakdown of the blood–follicle barrier (McClure *et al*. 1994, Irving-Rodgers *et al*. 2002), allowing IαI and PαI to diffuse into pre-ovulatory follicles where the HC components are incorporated into the cumulus ECM (Chen *et al*. 1996). In cumulus expansion, the HCs are transferred from IαI and PαI onto HA to form covalent HC·HA complexes; this process is catalysed by TSG-6 and occurs via TSG-6·HC intermediates (Rugg *et al*. 2005, Sanggaard *et al*. 2008). Mice that are deficient in the expression of either *bikunin* (and hence unable to assemble IαI) or *Tnfaip6* fail to support COC expansion (Sato *et al*. 2001, Zhuo *et al*. 2001, Fulop *et al*. 2003), indicating that the covalent modification of HA with HCs is essential for the assembly and cross linking of a stable cumulus ECM.

TSG-6 participates in multiple ECM remodelling processes (Milner & Day 2003, Milner *et al*. 2006) and is secreted by CCs and mGCs in response to ovulatory stimuli (Fulop *et al*. 1997*b*, Yoshioka *et al*. 2000, Carrette *et al*. 2001, Mukhopadhyay *et al*. 2001). As noted above, TSG-6 binds covalently to HCs during the catalysis of HC·HA formation (Rugg *et al*. 2005, Sanggaard *et al*. 2005, 2008). TSG-6-deficient female mice lack HC·HA complexes and are severely sub-fertile, which has been attributed to an unstable cumulus ECM leading to an absence of cumulus expansion (Fulop *et al*. 2003). Although TSG-6 contains a link module domain (in common with most other HA-binding proteins (Day & Prestwich 2002, Higman *et al*. 2014)) and binds directly to HA (Kohda *et al*. 1996), it is unclear whether TSG-6 is incorporated into the cumulus ECM.

PTX3, which is also secreted by CCs and required for ECM formation, is a multimeric protein, belonging to the pentraxin superfamily (Garlanda *et al*. 2005, Inforzato *et al*. 2010). COCs of PTX3-deficient mice have disorganised CC layers and females are sterile (Salustri *et al*. 2004). PTX3 has been found to interact with HCs (Scarchilli *et al*. 2007, Ievoli *et al*. 2011) and to play a key role in the organisation and cross-linking of HC·HA (Baranova *et al*. 2014).

As described previously, compromised COC expansion negatively affects female fertility in mice; in human IVF, the degree of cumulus expansion has been shown to be positively correlated with oocyte quality and thus fertilisation and implantation rates (Ng *et al*. 1999). However, the *C1galt1* mouse model is unique since it exhibits defective cumulus expansion, but fertility is not compromised (Williams & Stanley 2008, Grasa *et al*. 2014). These observations suggest that some aspects of cumulus expansion are redundant to successful fertilisation and the aim of this study was to identify these aspect(s).

**Figure 1:**
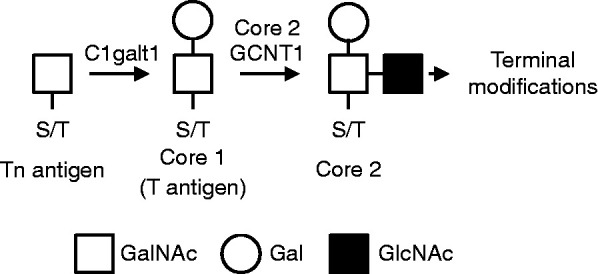
Action of C1galt1 in *O*-glycosylation. Core 1 β1,3-galactosyltransferase 1 (C1galt1) catalyses the addition of a galactose molecule to the Tn-antigen (*N*-acetylgalactosamine – serine/threonine) to form core 1 *O*-glycans, which are precursors to more complex *O*-glycans. Core 2 GCNT1 (β-1,6-*N*-acetylglucosaminyltransferase) extends core 1 *O*-glycans by the addition of *N*-acetylglucosamine to form core 2 *O*-glycans. In the *C1galt1* Mutant mice, oocytes do not produce C1galt1 and thus core 1-derived *O*-glycans are no longer synthesised on oocyte glycoproteins.

Eggs ovulated from *C1galt1* Mutant mice are surrounded by a cumulus mass that is denser and more resistant to hyaluronidase treatment compared with Control, indicating altered structure and function (Williams & Stanley 2008). Interestingly, *C1galt1 *Mutant mice exhibit increased fertility due to more follicles reaching the pre-ovulatory stage (Williams & Stanley 2008, Grasa *et al*. 2014). *C1galt1* encodes the glycosyltransferase T-synthase, also known as core 1 β1,3-galactosyltransferase (C1galt1), which is responsible for the synthesis of core 1-derived *O*-glycans (Fig. 1). *O*-glycosylation is a common post-translational modification and has important implications in determining the structure and function of glycoproteins. *O*-glycans have been shown to be important in receptor signalling (Stanley 2011), cell–cell interaction (Yago *et al*. 2010), cell–matrix interaction (Tian *et al*. 2012), and can provide protective roles for glycoproteins against proteolytic degradation (Pan *et al*. 2014). In the *C1galt1* Mutant, use of the ZP3*Cre* transgene enables deletion of *C1galt1* (and hence a lack of core 1-derived *O*-glycans on glycoproteins) specifically in oocytes from the primary follicle stage onwards (Philpott *et al*. 1987). The effects of oocyte-generated core 1-derived *O*-glycans, including those of OSFs, on surrounding CCs have not been investigated and therefore the *C1galt1* Mutant mouse provides a good model to investigate the role of these glycans on cumulus function.

Therefore, on the basis that the cumulus expansion defect in *C1galt1* Mutant mice does not lead to a respective compromise in subsequent fertility (as opposed to other mouse models with cumulus defects), our first hypothesis was that the altered cumulus mass was due to molecular changes in the cumulus ECM. Changes in any of the cumulus molecules would indicate either redundancy or plasticity in the function of the cumulus complex. In addition, considering the importance of the different cumulus ECM molecules (evident from the knock-out mouse models described previously) our novel intra-follicular approach, comparing ECM molecules of individual COCs, enabled us to determine the degree of correlation between these molecules in Control cumulus complexes.

In this study, we demonstrate that the modified cumulus matrix of *C1galt1* Mutant COCs results predominantly from the reduced Mutant cumulus size brought about by fewer CCs with additional minor changes in cumulus ECM proteins. These data have wider implications in the field of assisted reproductive technologies (ARTs) since selection of developmentally competent eggs should not be judged solely by the size of the cumulus complex and the number of CCs surrounding an egg. In addition, using this novel method of correlating the levels of cumulus ECM molecules within individual cumulus complexes, we provide evidence that considerable variations exist in the composition of the cumulus ECM, which are tolerated without adverse effects on fertility in both the Control and the Mutant, as long as all components are present above a threshold level.

## Materials and methods

### Animals and hormone treatments

*C1galt1* ^*FF*^:ZP3*Cre* male mice (*Mus musculus*) were mated with *C1galt1* ^*FF*^ female mice to generate *C1galt1* ^*FF*^:ZP3*Cre* (Mutant) and *C1galt1* ^*FF*^ (Control) female mice (Williams *et al*. 2007). Mice were kept in a 12 h light:12 h darkness cycle with lights on at 0700 h. For superovulation, mice were injected intraperitoneally with 5 IU of pregnant mare serum gonadotrophin (PMSG, Biosupply, Bradford, UK) at 1600 h and, 48 h later, with 5 IU of human chorionic gonadotrophin (hCG; Chorulon, Biosupply). All experiments were approved by the Home Office and the Clinical Medical Local Ethical Review Committee.

### Genotyping

Mice were genotyped using protocols adapted from Williams *et al*. (2007). Each 25 μl PCR contained 2.5 μl of 10× PCR buffer (Bioline, London, UK), 0.75 μl of 50 mM MgCl_2_ solution (Bioline), 0.5 μl of 10 mM dNTP (Roche, Mannheim, Germany), 0.5 μl of each primer (Eurogentec, Liege Science Park, Seraing, Belgium), 1 μl of genomic DNA (ear) and 0.15 μl of Taq polymerase (Bioline) for the detection of floxed *C1galt1* or 0.5 μl of Taq polymerase for the detection of the *Cre* transgene. The primers used to detect the *C1galt1* floxed allele were either TS1 (Williams *et al*. 2007) and TS8 (TCTGCATTGAAGTTCATCTGT) or FB33 and FB34 (Batista *et al*. 2012) and the *Cre* transgene was detected using primers PS502 and PS607 (Shi *et al*. 2004).

### Ovary collection and histology

Ovaries from 8- to 9-week-old mice were dissected 9 h post-hCG, fixed in 10% buffered formalin (Sigma–Aldrich, Dorset, UK) for 8 h and washed in 70% ethanol. The ovaries were embedded in paraffin, sectioned at 5 μm and mounted on glass slides.

### Histochemistry and immunohistochemistry

The sections were deparaffinised and rehydrated. Antigen retrieval was performed to enable detection of TSG-6, Ki-67 and pSMAD2 (low pH solution, Vector Labs, Peterborough, UK) and pSMAD1/5/8 (0.01 M citrate buffer). Endogenous peroxidase was blocked with 3% H_2_O_2_ (Fisher Scientific, Loughborough, UK) in PBS for 5 min. The sections were blocked with 2% FCS (Sigma–Aldrich) in PBS for 1 h before HA detection, or with 1.5% normal goat serum (NGS, Vectastain ABC Kit, Vector Labs) in TBS for 1 h before TSG-6, Ki-67 and pSMAD2 detection, or with 10% dry milk (Alcafe, Reading, UK) in PBS for 1 h before PTX detection, or with 5% dry milk in PBS for 2 h before HC detection, or with 5% BSA (Fisher Scientific) in PBS for 1 h before pSMAD1/5/8 detection. The sections were then incubated with either 0.25 mg/ml biotinylated HA binding protein (bHABP (Clark *et al*. 2011), Seikagaku, Tokyo, Japan) at 1:50, or rabbit anti-mouse TSG-6 polyclonal anti-sera (Carrette *et al*. 2001) at 1:150, or 1 mg/ml rabbit anti-human PTX3 polyclonal antibody ((Scarchilli *et al*. 2007), generously supplied by Dr Antonio Inforzato) at 1:200, or 7.6 mg/ml rabbit anti-human IαI/PαI polyclonal antibody at 1:100 (to detect HCs) ((Carrette *et al*. 2001, Mukhopadhyay *et al*. 2001); Dako, Glostrup, Denmark), or rabbit anti-Ki-67 antibody (Abcam, Cambridge, UK) at 1:100, or rabbit anti-pSMAD2 polyclonal antibody (0.25 mg/ml; Life Technologies, Invitrogen, Paisley, UK) at 1:100, or anti-pSMAD1/5/8 polyclonal antibody (Cell Signalling, Beverly, MA, USA) at 1:250; all reagents were diluted in their respective blocking solution and incubated for 2 h at room temperature (HA, Ki-67 and pSMAD2) or at 4 °C overnight (TSG-6, PTX3, HC and pSMAD1/5/8). The specificity of anti-pSMAD1/5/8 was determined by western blot analysis of BMP2-treated HeLa and MEF cells (Cell Signalling), anti-pSMAD2 was shown to be specific by western blot analysis of TGF-β-stimulated HepG2 cells (Life Technologies), immunogen affinity-purified anti-Ki-67 was assessed using immunohistochemistry with positive control tissue (Abcam; Chen *et al*. 2014, Nenicu *et al*. 2014). The specifities of the anti-IαI (Carrette *et al*. 2001, Mukhopadhyay *et al*. 2001, Salustri *et al*. 2004), anti-TSG-6 (Carrette *et al*. 2001, Mukhopadhyay *et al*. 2001) and anti-PTX3 reagents (Salustri *et al*. 2004) have all been demonstrated by western blot analysis in the context of murine COC extracts.

All immunohistochemistry slides were incubated with biotinylated anti-rabbit IgG secondary antibody (Vectastain ABC Elite Kit, Vector Labs) for 1 h at room temperature, followed by ABC solution (Vectastain ABC Elite Kit) for 30 min at room temperature. Antigen-specific detection was revealed using a DAB Kit (Vector Labs). The slides were then dehydrated and mounted with Depex (VWR, Leicestershire, UK) and imaged using the same light microscope (Leica DM 2500, Microscope services Ltd, Woodstock, UK). Experiments to detect HA and protein antigens using bHABP or antibodies, respectively, were all performed a minimum of three times.

### Characterisation of cumulus complex

Molecules detected in CCs and cumulus ECM were quantified using ImageJ Software (National Institutes of Health, Bethesda, MD, USA). In ImageJ, each pixel is given an intensity value from 0 (black) to 255 (white); based on this, total pixel intensity and mean pixel intensity are calculated. The values for total pixel intensity, number of pixels, and mean pixel intensity for each cumulus complex were all calculated and exported from ImageJ. The pixel values were then inverted, therefore 0=white and 255=black, to facilitate data interpretation. Finally, mean pixel intensity was expressed as a percentage (i.e. scale 0–100). To normalise to CC numbers, the value of total pixel intensity was simply divided by CC numbers.

**Figure 2:**
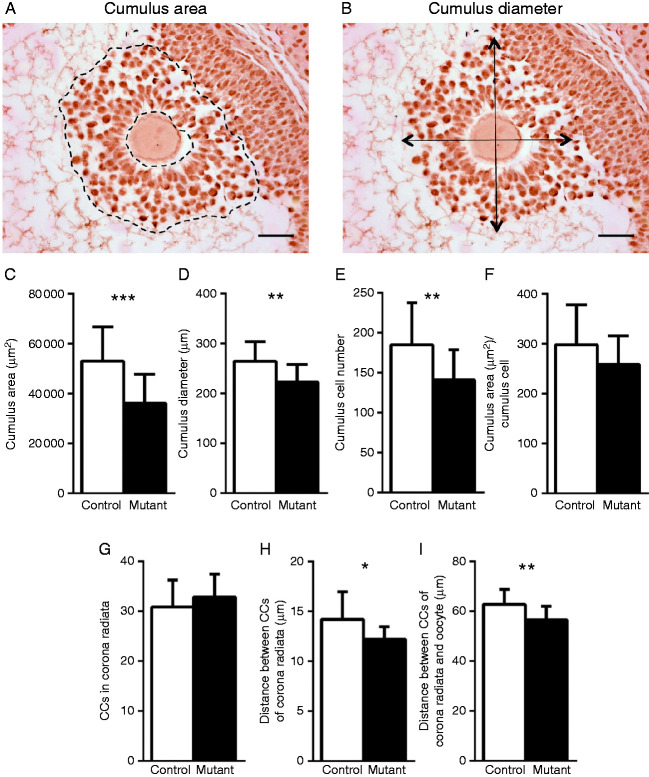
Oocyte-derived *O*-glycans modify cumulus expansion. The central section through each oocyte was selected and the size of the cumulus complex assessed by determining the area occupied by the CCs (A) and by averaging the two largest perpendicular diameters of the COC (B). The following values were determined for Control and Mutant COCs: size of the cumulus area in COCs (C), average diameter of COCs (D), total CC number making up the cumulus complex (E), density of CC distribution in COCs (F), number of CCs making up corona radiata (G), average distance between adjacent CCs in the corona radiata (H) and average distance between corona radiata CCs and the centre of the oocyte (I). Data are presented as mean values±s.d. (C, D, E and F): *n*=16 Control and *n*=22 Mutant COCs. (G, H and I): *n*=13 Control and *n*=29 Mutant COCs. *P<0.05; **P<0.01 and ***P<0.001. Scale bar: 50 μm.

To analyse the size of cumulus complexes, the section closest to the centre of the oocyte was chosen. Cumulus area was quantified by selecting the space occupied by CCs (Fig. 2A), and average cumulus diameter was determined by averaging the two largest perpendicular diameters in the cumulus complex (Fig. 2B). To count the total number of CCs in each COC and the number of CCs in the corona radiata, the centremost section of the COC was counterstained with haematoxylin (Shandon Gill 2 Haematoxylin, Fisher Scientific) and the number of CCs was determined using the count tool in ImageJ. To measure the distance between each corona radiata CC and the distance between corona radiata CCs and the oocyte, the straight-line tool in ImageJ was used. The numbers of complexes analysed are given in respective figure legends.

### Statistical analysis

All bar graphs values are presented as mean±s.d. and data were analysed using Student's *t*-test (GraphPad Prism Software, Inc., San Diego, CA, USA). A *P* value of <0.05 was considered to be statistically significant. For correlations of ECM molecules and cumulus expansion, the coefficient of determination (*r* ^2^) was calculated (GraphPad Prism) to establish the degree of association between the variables. An *r* ^2^ value of >0.8 was considered to indicate a strong association.

## Results

### Cumulus expansion in the *C1galt1* Mutant is reduced

Eggs from superovulated *C1galt1* Mutant female mice are surrounded by modified cumulus masses compared with Controls (Williams & Stanley 2008). To characterise and quantify the Mutant phenotype in expanded COCs, *C1galt1* Mutant and Control females were induced to ovulate using exogenous gonadotrophins. Ovaries were collected 9 h post-hCG and sections through the centre of each oocyte were selected for subsequent analysis (oocyte diameter did not differ between Control and Mutant follicles, data not shown). Analysis of these sections revealed that cumulus mass area (Fig. 2A) and diameter (Fig. 2B) were both significantly decreased in Mutant follicles (∼32% decrease; Fig. 2C and ∼16% decrease; Fig. 2D respectively). Cumulus cell (CC) counts further revealed that the reduced cumulus mass area contained a significantly smaller number of CCs in the Mutant (∼24% fewer) compared with Controls (Fig. 2E). Therefore, although the amount of space occupied by each CC (i.e. density) did not differ in Controls and Mutants, there was a ∼13% decrease in average area per CC in the Mutant, reflecting a non-significant increase in density (Fig. 2F).

The innermost layer of the cumulus mass is known as the corona radiata. The number of CCs in the corona radiata was similar in Mutant and Control (Fig. 2G). However, the distance between each corona radiata CC and the distance between corona radiata CCs and the oocyte were both decreased in the Mutant (Fig. 2H and I respectively).

### Cumulus ECM composition is altered in the *C1galt1* Mutant

**Figure 3:**
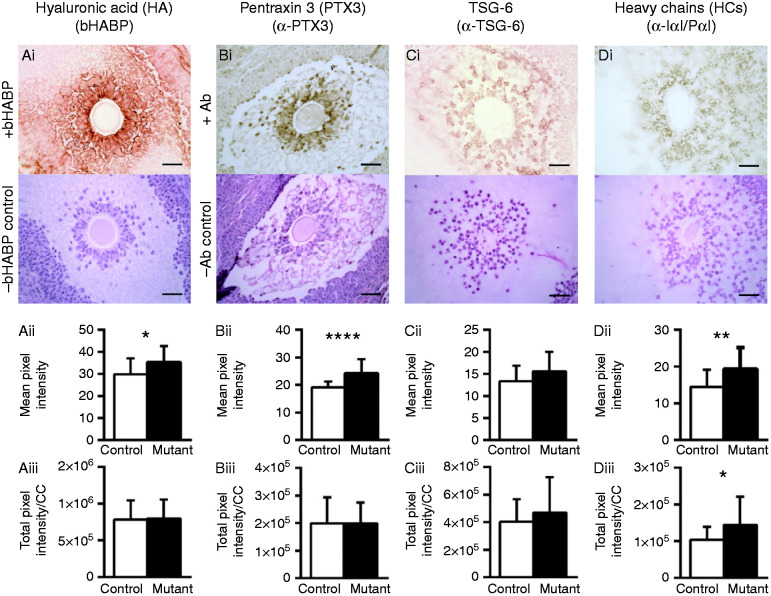
Localisation and quantification of HA, PTX3, TSG-6 and IαI in preovulatory cumulus masses. Localisations of (Ai) HA, (Bi) PTX3, (Ci) TSG-6, (Di) HCs were determined in preovulatory follicles (molecule used for detection in brackets). Respective mean pixel intensities (Aii), (Bii), (Cii), (Dii) and total pixel intensities normalised to CC number (Aiii), (Biii), (Ciii) and (Diii) were determined for each of the matrix components. The upper panels of (Ai), (Bi), (Ci) and (Di) show representative images from COCs stained with bHABP or a protein-specific antibody; lower panels show sections counterstained with haematoxylin in the absence of any bHABP or primary antibody (i.e. only secondary detection reagents were applied). Data are presented as mean values±s.d. (Aii and Aiii): *n*=17 Control and *n*=21 Mutant. (Bii and Biii): *n*=17 Control and *n*=23 Mutant. (Cii and Ciii): *n*=20 Control and *n*=20 Mutant. (Dii and Diii): *n*=16 Control and *n*=21 Mutant. *P<0.05; **P<0.01 and ****P<0.0001. Scale bar: 50 μm. Ab, antibody.

Having determined that *C1galt1* Mutant COCs have smaller cumulus masses, we investigated the molecular origin of this phenotype by analysing the cumulus ECM composition. HA was detected throughout the cumulus ECM and also around the peripheral mGCs closest to the CCs (Fig. 3Ai). Quantification revealed that even though the mean intensity of HA staining in the COC was increased in the Mutant (Fig. 3Aii), when normalised to CC number the stain density was similar in Control and Mutant COCs (Fig. 3Aiii). PTX3 was also detected (Fig. 3Bi) and although mean staining intensity was increased in the Mutant (Fig. 3Bii), intensities per CC were similar in Control and Mutant (Fig. 3Biii). TSG-6 was detected surrounding the CCs (Fig. 3Ci), and the staining intensities were comparable in Mutants and Controls (Fig. 3Cii and iii). IαI and PαI enter ovarian follicles from serum and the HC components become covalently attached to HA. The presence of HCs in the cumulus mass was assessed using an antibody which detects bikunin and the HCs of IαI and PαI; this analysis is hereafter referred to as HC (Fig. 3Di). HC detection revealed a similar pattern to that seen for HA, with an increase in staining intensity in Mutants compared with Controls (Fig. 3Dii). However, in contrast to the other matrix components investigated here, HCs were found to be more densely distributed in Mutant cumulus ECM compared with Controls (Fig. 3Diii). Overall, these results indicate that the CCs surrounding the Mutant oocytes are not functionally different compared with Controls, as they produce normal levels of ECM components. However, there is evidence of molecular and therefore structural differences between the cumulus matrix in Mutant and Control mice.

### Quantification of cumulus intracellular molecules

**Figure 4:**
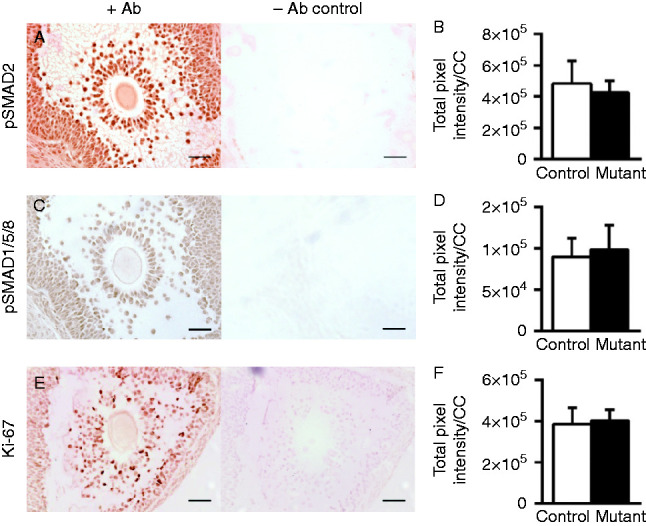
Localisation and quantification of pSMAD2, pSMAD1/5/8 and Ki-67 in preovulatory cumulus masses. Localisations of (A) pSMAD2, (C) pSMAD1/5/8, (E) Ki-67 and respective total pixel intensity normalised to CC number (B), (D) and (F) were determined for Mutant and Control preovulatory follicles. Left panels of (A), (C) and (E) show representative images from COCs stained with each of the protein-specific antibodies; right panels show sections without addition of primary antibody, showing no visible DAB staining. Data are presented as mean values±s.d. (B): *n*=15 Control and *n*=18 Mutant COCs. (D): *n*=19 Control and *n*=20 Mutant COCs. (F): *n*=16 Control and *n*=19 Mutant COCs. Scale bar: 50 μm.

Cumulus expansion requires OSF action on the CCs. As the *C1galt1* Mutant has an oocyte-specific deletion which affects secreted core 1-derived *O*-glycoproteins, we hypothesised that this mutation might directly or indirectly affect the function of one or more OSFs. To assess the function of the OSF involved in cumulus expansion, we examined intracellular signalling pathways activated in response to TGF-β ligands (key OSFs are members of the TGF-β superfamily); i.e., the SMAD2 and SMAD1/5/8 pathways. Localisation of pSMAD2 was cell-associated as expected (Fig. 4A) and normalisation of the stain to CC numbers revealed similar levels in Controls and Mutants (Fig. 4B). In addition, the levels of pSMAD1/5/8, which was also cell-associated (Fig. 4C), did not differ between Controls and Mutants (Fig. 4D).

As modified cumulus expansion in the *C1galt1* Mutant is not associated with changes in the ability of CCs to deposit ECM, we investigated whether the proliferative potential of CCs was altered in the Mutant leading to changes in CC counts. Localisation and quantification of Ki-67, a nuclear marker of proliferation (Fig. 4E), revealed that levels of Ki-67 were similar in Control and Mutant CCs (Fig. 4F) indicating that cell proliferation was unaltered at 9 h post-hCG.

### Correlations between cumulus expansion and cumulus molecules

Although the requirement for HA, PTX3, TSG-6 and HCs in cumulus expansion and their inter-dependence during ECM deposition are well described, the relationship between the available concentrations of these molecules and the extent of expansion have not been analysed in detail. In this study, we tested whether there was any correlation between the amount of each molecule present in the cumulus mass (as determined by staining intensity) and the cumulus area (Supplementary Figure 1, see section on supplementary data given at the end of this article). Surprisingly, in Controls, the extent of cumulus expansion did not correlate with the quantity per CC of HA, TSG-6, PTX3 or HCs (Supplementary Figure 1A, B, C and D) nor did levels of OSF-induced pSMAD2 per CC correlate with cumulus size (Supplementary Figure 1E). Furthermore, in *C1galt1* Mutant mice, the lack of oocyte glycoproteins with core 1-derived *O*-glycans did not alter the relationship between ECM molecules or pSMAD2 with cumulus size, as no correlations were observed (Supplementary Figure 1F, G, H, I and J) similar to Controls.

### Correlations between different cumulus molecules

In light of the interdependencies between ECM components during cumulus expansion (Fulop *et al*. 2003, Salustri *et al*. 2004, Scarchilli *et al*. 2007), the relationships between the quantities of these molecules within individual COCs were also investigated (Supplementary Figure 2, see section on supplementary data given at the end of this article). Somewhat surprisingly, no correlation was found between any combination of cumulus ECM proteins in either Control or Mutant revealing unexpected flexibility in the system. As activation of the SMAD2/3 pathway in CCs is essential for cumulus expansion, we also investigated the relationship between the levels of CC-derived ECM components and the levels of pSMAD2 in CCs. Again, no correlations were observed in either Controls (Supplementary Figure 3A, B and C) or Mutants (Supplementary Figure 3D, E and F).

## Discussion

Cumulus mass expansion has important roles in oocyte development, ovulation and is believed to facilitate the transfer of ovulated eggs to the oviduct. Indeed, historically, CC numbers surrounding eggs for human IVF have been thought to be a useful marker of implantation potential (Gregory 1998). Cumulus expansion in the preovulatory follicles of mice requires paracrine signals from the oocyte, which act on CCs to promote the formation of a HA-rich ECM. Even though the programming of the granulosa cells surrounding the oocyte to differentiate into CCs is understood to be dependent on OSFs, the specific OSF(s) critical for regulating cumulus expansion in mice have not yet been determined.

Our results reveal that reduced cumulus size does not prevent ovulation and subsequent fertilisation, as these processes are not compromised in the *C1galt1 *Mutant despite a ∼32% decrease in cumulus size compared with Controls. These data suggest that there is a minimum size of cumulus required, below which ovulation and fertilisation are negatively affected. If this is the case, then the extent of cumulus matrix expansion within *C1galt1* Mutant follicles is sufficient to support ovulation and fertilisation. We also present novel analysis of the associations between the different cumulus ECM molecules by determining the levels of essential cumulus matrix molecules (i.e. HA, HCs, TSG-6 and PTX3) within individual COCs. These analyses reveal that there are no strong correlations either between the amount of any one of these ECM molecules and the size of the cumulus mass or between the relative levels of any of the matrix components in both Control and Mutant COCs. This suggests a highly flexible system whereby the relative amounts of HA, HCs, TSG-6 and PTX3 can vary quite substantially but can still form a functional matrix provided that they are all present at, or above, the minimum level required. Furthermore, the degree of cumulus expansion does not predict the respective levels of cumulus ECM molecules.

The role of the cumulus complex in supporting oocyte maturation (De Matos *et al*. 2008, Lu *et al*. 2013) has been identified as an important factor in determining the success of some human-ART methods (e.g. *in vitro* maturation (IVM)). The low success rate of ARTs (IVM <35% (Ellenbogen *et al*. 2014) and IVF <40% (Hogue 2002)) is partly attributed to the selection of eggs that, despite possessing a normal complement of chromosomes, have other impairments. Therefore, the development of objective criteria to define oocyte quality is of great importance. It has been suggested that CC assessment can be an informative predictor of oocyte developmental potential, because CC proliferative potential has been positively correlated with pregnancy rates (Gregory 1998, Khurana & Niemann 2000). The results presented in this report indicate that the ∼23% decrease in CC number associated with oocytes in *C1galt1* Mutants is not detrimental to fertilisation and implantation and therefore a reduction in this magnitude in CC number is not a reliable assessment to predict oocyte developmental potential. As a result, a partially expanded cumulus complex in human ARTs may not be the best indicator of oocyte quality.

**Figure 5:**
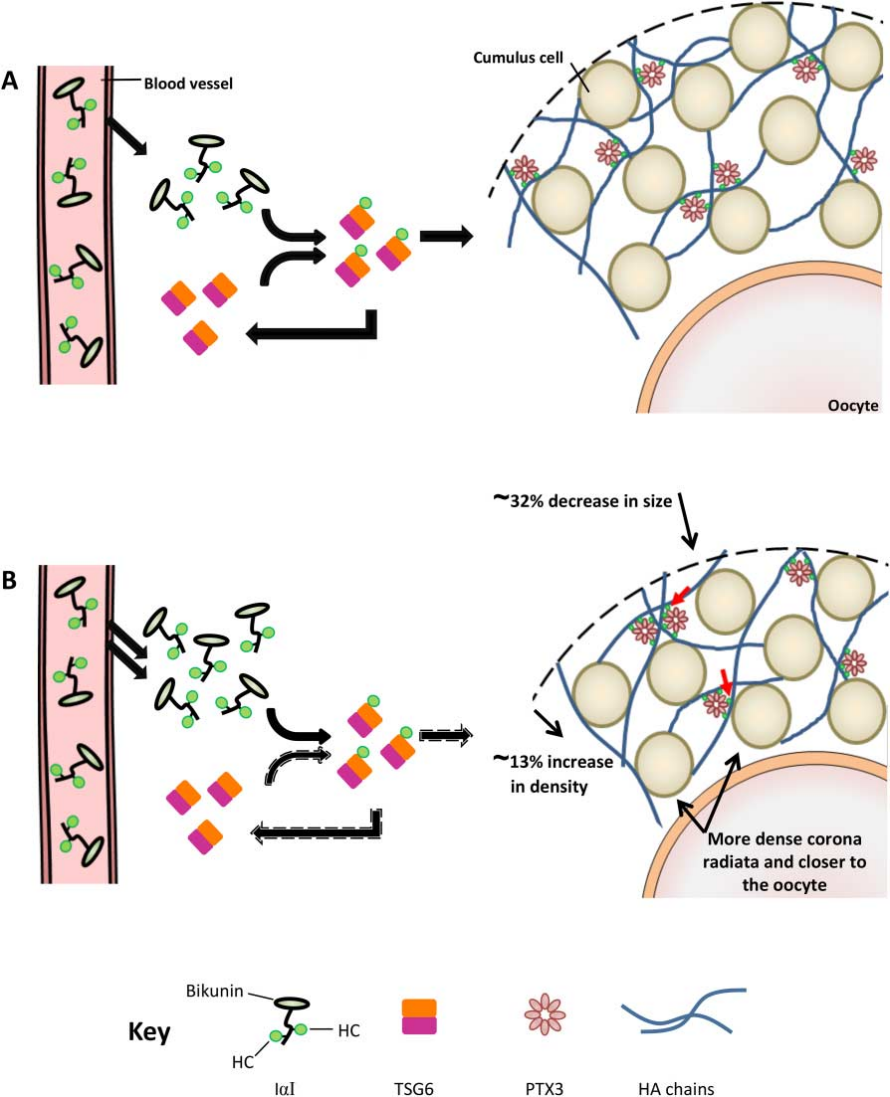
Proposed model of modified cumulus expansion in *C1galt1* Mutant mice. (A) Schematic model of the interactions between HA and HA-binding proteins in ECM from Control COCs. IαI family proteins are transported by blood and following the ovulatory LH surge, they diffuse into the preovulatory follicle. TSG-6 molecules, secreted by cumulus cells, act as catalysts in the transfer of the heavy chains (HCs) components of IαI onto HA chains. HA is the structural backbone of cumulus ECM and interactions with the multimeric PTX3 and HC enable HA chain cross linking. (B) The cumulus mass of Mutant COCs occupies ∼32% less area compared with the Control (Fig. 2C), and also contains fewer CCs (Fig. 2E). In addition, the area per CC in the Mutant is ∼13% less than Controls (Fig. 2F). The cumulus ECM of the Mutant contains increased amounts of HCs per CC (red arrows; Fig. 3Diii), while levels of HA, PTX3 and TSG-6 per CC remain similar to Controls (Fig. 3Aiii, Biii and Ciii). The modified basal lamina of Mutant follicles (Christensen *et al*. 2014) may allow the influx of more IαI molecules during follicle development and the periovulatory period (double arrows from blood vessel); this could result in increased transfer of HCs onto HA (arrows with broken border), resulting in a higher degree of HA chain cross linking. As a result, the Mutant develops a smaller, denser, cumulus mass compared with Controls. The relative sizes of the molecules and cells and the relative sizes of each of the components of IαI are not to scale, as HCs are bigger compared with the Bikunin component.

To investigate the origin of the reduced cumulus size observed in the *C1galt1* Mutant, we examined two parameters that underlie the formation of the cumulus matrix during the periovulatory period: i) the number of CCs that make up the CC complex and ii) the ability of each individual CC to produce cumulus ECM molecules. Mutant COCs were shown to occupy ∼32% less area compared with the Control, which is accompanied by fewer CCs in the entire complex. Furthermore, the corona radiata CCs in the Mutant were more tightly packed and were also closer to the oocyte compared with Control indicating aberrant expansion. In addition, the area occupied per CC in the Mutant is ∼13% less than Controls, making the Mutant COC ∼13% more dense. As a result of this, the mean intensity of HA and PTX3 molecules was higher in the Mutant COC, although when analysed per CC, the intensity of these two molecules was similar between Control and Mutant, indicating that each individual CC in the Mutant functions as Control. Interestingly, the levels of HCs detected, which are the only cumulus ECM component not produced within the follicle, were increased in Mutants compared with Controls. An increased production of IαI, and thus HC, by the liver due to the oocyte modification is unlikely. However, it has been observed that the basal lamina of Mutant follicles is altered during follicle development (Christensen *et al*. 2014). Therefore, in these mice the basal lamina may be permeable to IαI even in the absence of an ovulatory stimulus, such that the intrafollicular presence of IαI, and hence HCs, is elevated compared with Controls during the periovulatory period. Increased levels of HCs could result in more extensive HA cross linking (Baranova *et al*. 2014) and hence a more compact cumulus matrix. Therefore, although CCs in *C1galt1* Mutant mice appear functionally normal, as demonstrated by cumulus intracellular signalling pathways and the ability of CCs to produce ECM components, the combined effects of fewer CCs and more HCs could result in the production of a cumulus matrix with altered organisation in *C1galt1 *Mutant mice (see proposed model in Fig. 5).

The decreased number of CCs in Mutant preovulatory follicles suggests that these cells have an altered proliferative potential, but there was no difference in Ki-67 levels between Control and Mutant. However, the reduced CC number in Mutant follicles suggests that: i) there was altered proliferation of CCs in earlier stages of cumulus expansion, or ii) there were fewer somatic cells associated with the oocyte from the outset, or iii) CC apoptosis is elevated in the Mutant resulting in fewer CCs at 9 h post-hCG. The second hypothesis is consistent with the characteristics of Booroola sheep that exhibit increased fertility (similar to *C1galt1* Mutant mice) resulting from heterozygosity of a mutation in bone morphogenetic protein receptor 1B (BMPR1B; a receptor for TGF-β superfamily molecules). In these sheep, the increased number of preovulatory follicles is accompanied by a smaller number of granulosa cells per follicle, resulting in fewer cells contributing to the cumulus complex (McNatty & Henderson 1987). However, as SMAD signalling (activated by TGF-β superfamily molecules) was unaltered in *C1galt1* Mutant mice, it is unlikely that TGF-β signalling is modified at this stage by the oocyte-generated core 1-derived *O*-glycans, including those of OSFs. This does not rule out changes in COC signalling at earlier stages of Mutant follicle development which may be the origin of the reduced number of CCs.

In conclusion, the absence of core 1-derived *O*-glycans from oocyte-expressed glycoproteins has effects on the whole follicle that are evident from i) greater levels of HCs of IαI and Pαl in the follicle and ii) altered numbers of CCs. This highlights the critical role of the oocyte in follicle development. The effects of *C1galt1* deletion on the cumulus mass surrounding the oocyte could be a direct result of changes in the OSFs that determine the proliferative potential of CCs or an indirect outcome relating to the effects of OSFs on EGF-ligands/EGF-receptors, whereby if these are altered, the LH signal is not properly transmitted to CCs. The observation that mouse COCs with reduced CC numbers can function normally without compromising the developmental potential of oocytes is intriguing and raises the question of why do Control COCs have an apparent excess of CCs? In addition, it will be interesting to investigate whether this observed CC redundancy in mouse COCs applies to human COCs, in which case, assessment of the cumulus complex as an indication of oocyte quality for IVF needs to be used with caution. Finally, the lack of any strong correlations between the levels of different ECM molecules relative to each other or to the size of the cumulus mass indicates that, providing the minimum requirements for matrix formation are met, this system possesses a high degree of flexibility. It remains to be determined whether the specific expression patterns of individual ECM molecules by human CCs might be predictive of oocyte quality.

## Supplementary data

This is linked to the online version of the paper at http://dx.doi.org/10.1530/REP-14-0503.
